# Comparative Physicochemical Analysis among 1,4-Butanediol Diglycidyl Ether Cross-Linked Hyaluronic Acid Dermal Fillers

**DOI:** 10.3390/gels7030139

**Published:** 2021-09-11

**Authors:** Nicola Zerbinati, Sabrina Sommatis, Cristina Maccario, Maria Chiara Capillo, Giulia Grimaldi, Giuseppe Alonci, Raffaele Rauso, Stefania Guida, Roberto Mocchi

**Affiliations:** 1 Department of Medicine and Surgery, University of Insubria, 21100 Varese, Italy; nicola.zerbinati@uninsubria.it; 2 UB-CARE S.r.l.-Spin-off University of Pavia, 27100 Pavia, Italy; sabrina.sommatis@ub-careitaly.it (S.S.); cristina.maccario@ub-careitaly.it (C.M.); mariachiara.capillo@ub-careitaly.it (M.C.C.); research@ub-careitaly.it (G.G.); 3 Department of Research and Development, Matex Lab Switzerland SA, 1228 Geneve, Switzerland; giuseppe.alonci@neauvia.com; 4 Maxillofacial Surgery Unit, University of Campania “Luigi Vanvitelli”, 81100 Caserta, Italy; dr.raffaele.rauso@gmail.com; 5 Dermatology Unit, Department of Surgical, Medical, Dental and Morphological Sciences Related to Transplant, Oncology and Regenerative Medicine, University of Modena and Reggio Emilia, 41124 Modena, Italy; stefania.guida@unimore.it

**Keywords:** hyaluronic acid, rheology, matrix structure, BDDE, injectable HA dermal fillers, aesthetic application, polymers cross-linking technology

## Abstract

(1) Background: Injectable hyaluronic acid (HA) dermal fillers are used in several chirurgical practices and in aesthetic medicine. HA filler stability can be enhanced through different cross-linking technologies; one of the most frequently cross-linker used is 1,4-butanediol diglycidyl ether (BDDE), also present in the HA-BDDE dermal filler family of the company Matex Lab S.p.A. (Brindisi, Italy). Our overview is focused on their characterization, drawing a correlation between matrix structure, rheological and physicochemical properties related to their cross-linking technologies. (2) Methods: Four different injectable HA hydrogels were characterized through optical microscopic examination and rheological behavior investigation. (3) Results: The cross-linked HA dermal fillers showed a fibrous “spiderweb-like” matrix structure and an elastic and solid-like profile. (4) Conclusions: The comparative analysis represents a preliminary characterization of these injectable medical devices in order to identify their best field of application.

## 1. Introduction

**Figure 1:**
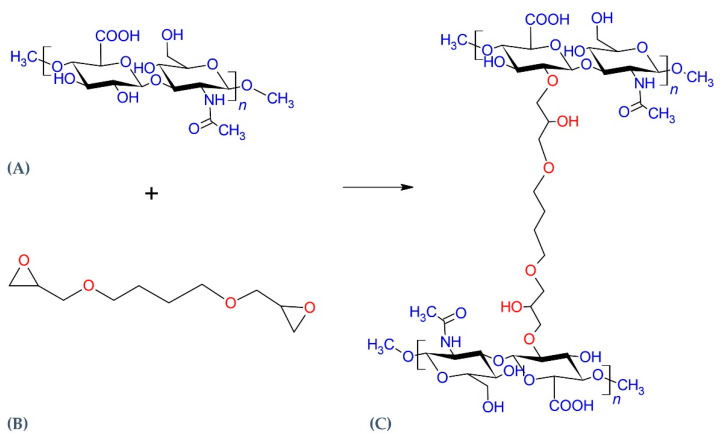
Schematic illustration of cross-linking reaction between hyaluronic acid (HA) (**A**) and the (1,4-butanediol diglycidyl ether) BDDE cross-linking agent (**B**). Under alkaline conditions the BDDE epoxy groups react with the hydroxyl HA group, leading to ether bond formation (**C**). Image obtained with ChemSketch 2020.1.2 (Advanced Chemistry Development, Inc., Toronto, CA, Canada).

Hyaluronic acid (HA) is a naturally occurring glycosaminoglycan (GAG), a linear polymer composed of alternating disaccharide units of D-glucuronic acid and N-acetyl-glucosamine, linked via β-1,4 and β-1,3 glycosidic bonds [1]. HA unbranched single-chain polymers vary with respect to molecular weight, ranging from 10^5^ to 10^7^ kDa. HA is a main component of the extracellular matrix (ECM), together with collagen and elastin fibers; its main physiological features are the high hydration ability (water sorption and retention), related to its hydrophilic nature, and the high lubricant ability. HA plays a key role in different biological processes, as cell signaling and wound repair [2]. Its peculiar physical and mechanical properties, as well as its high biocompatibility, make the HA polymer an ideal viscoelastic material for ophthalmic and orthopedic surgery, soft tissue augmentation, facial rejuvenation and drug delivery [3]. Recently, a sensitive challenge has been to exploit HA-based nanocapsules as carriers for the delivery of hydrophobic anti-cancer compounds across the cancer cell’s membrane and improving some of their properties, such as circulation time and cellular uptake [4]. HA is structurally homogenous across different species, and for industrial application it is usually extracted from animal tissues or bacterial strains. These features make it a biorenewable resource for nanocomposite materials that can be produced easily by several methods, such as melt mixing, in situ polymerization, solution mixing, precipitation, sol–gel processes etc. Composites consist of at least two components associated in a continuous matrix phase and a non-continuous reinforcement material to enhance the qualities of each component, and nowadays represent the most important materials of modern technology. The HA chain is consists of carboxylic groups, hydroxyl groups, and -NHCOCH_3_ groups, all active functional sites suitable for covalent modification, such as amination or esterification [5,6,7]. The HA content in the body is subject to a rapid physiological turnover, which sets limits for clinical application of exogenous, unmodified HA. Recently, the stability of HA soft-tissue fillers was improved by chemical cross-linking of the HA, in order to optimize the products’ biophysical and mechanical properties, promoting their long-lasting effect in vivo [8]. Most of them are manufactured using 1,4-butanediol diglycidyl ether (BDDE) as the cross-linking agent (Figure 1). The cross-linking process improves the in vivo duration and the biophysical properties of the product transforming the linear HA chains into a three-dimensional network [9]. This process, performed according to different manufacturing technologies, leads to hydrogels (hydrophilic polymeric network cross-linked via covalent or non-covalent bonds) with specific rheological properties and matrix structures [10,11]. In particular, the BDDE cross-linking takes place under alkaline conditions (pH > 7), because this condition allows the reaction of BDDE’s epoxide group and HA’s primary alcohol, forming an ether connection that has a greater stability than amide or ester bonds. The ether bonds guarantee a major clinical duration despite BDDE having a low toxicity, and is biodegradable [12]. The HA derivative has different physicochemical and rheological properties from the non-crosslinked HA, but most crosslinked filler maintains the biodegradability and biocompatibility of the native HA [13].

During past few years, rheological characterization of HA dermal fillers has played a key role in defining their mechanical behavior, promoting safe and good results during their clinical applications [14]. Rheology makes it possible to identify the physicochemical properties of the sample and the appropriate administration site and depth of injection through the characterization of the main representative parameters: the elastic modulus (G’), the viscous modulus (G’’), the complex modulus (G*), and the tangent of the phase angle (tan δ). Solids have G’ >> G”, i.e., the elastic component is much greater than the viscous one, while for fluids, the opposite is true, and G” > G’ [15]. Hydrogels are classified as viscoelastic materials, so they present both viscous and elastic behavior, depending on the conditions. However, it is generally acknowledged that gelation happens when G’ becomes greater than G”, because of the influence of the intermolecular forces, both covalent and non-covalent interactions [16]. A material can also behave as a gel under some conditions and as a viscous fluid under other conditions. Therefore, these parameters are useful for understanding which of these components prevails and how the material behave under stress [17,18]. For example, in pure, un-crosslinked HA solutions, G’ is higher than G” (tan δ < 1) at high frequencies (solid-like behavior), while at lower frequencies G” is higher than G’ (tan δ > 1, fluid-like behavior). On the contrary, covalently crosslinked HA fillers have a gel-like behavior at all frequencies, and usually tan δ < 0.5. The higher the value of G’, the greater the hydrogel stiffness, which confers to the material a lower susceptibility to deformation. When the values of G’ and G’’ are similar (tan δ ≈ 1), the material has an intermediate behavior between a solid and a liquid, and the mechanical strength is low. If G’’ is greater than G’, the lower elastic modulus with respect to the loss modulus describes a liquid-like material, rather than a gel [19]. Knowledge of rheological behavior helps researchers to evaluate how the hydrogels react and deform under the mechanical stress typical of the injection and after implantation. Likewise, rheology can guide clinicians in choosing the most appropriate product for a determinate body region, injection technique and desired effect [9]. For a better characterization of a HA hydrogel, the definition of its matrix structure is another peculiar feature related to the cross-linking process as well as to the ratio between HA and cross-linking agent (cross-linking degree). Three HA matrix structures have previously been characterized: a “spiderweb”-like structure, a particulate structure, and an intermediate structure. These differences can be detected by optical microscopy and make it possible to distinguish monodensified from polydensified hydrogels [20].

Our study was focused on the rheological and physicochemical properties of different HA-based dermal fillers cross-linked with BDDE and not cross-linked (Matex Lab S.p.A, Brindisi, Italy), in order to better underline and characterize the impact of the cross-linking process on the matrix structure organization and on the rheological behavior. Four hydrogels cross-linked with BDDE and with different HA content (Table 1) were characterized in comparison with an 18-mg/mL not cross-linked HA-based dermal filler.

## 2. Results and Discussion

### 2.1. Optical Microscopic Examination

**Figure 2:**
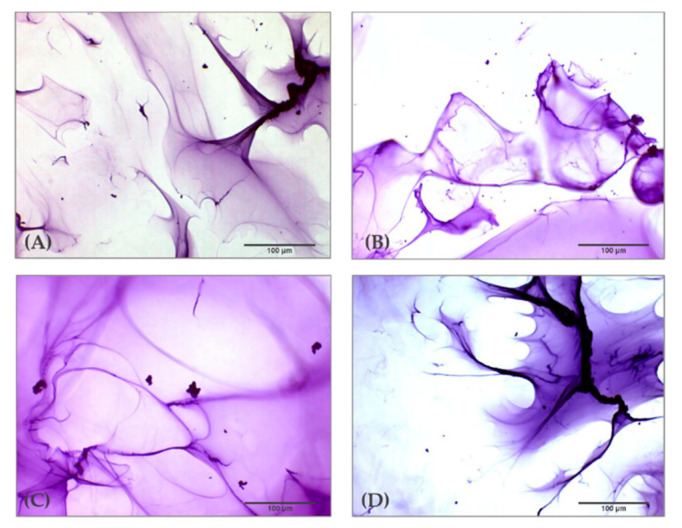
Optical microscopic examination (10X) of four HA dermal fillers cross-linked with BDDE after staining with toluidine blue 1%. (**A**) HA 22 mg/mL cross-linked with BDDE; (**B**) HA 24 mg/mL cross-linked with BDDE; (**C**) HA 26 mg/mL cross-linked with BDDE; (**D**) HA 28 mg/mL cross-linked with BDDE.

Observation of the tested HA-based hydrogels by optical microscopy resulted in the visualization of a peculiar matrix structure. In Figure 2 it is possible to observe the structure of the four HA hydrogels cross-linked with BDDE obtained with 10× magnification. The investigation demonstrates that the organization of microscopic areas of the HA hydrogels do not show remarkable differences among the dermal fillers despite the different concentrations of HA in their composition; rather, they a peculiar and homogenous fibrous matrix structure resembling a “spider web”. Furthermore, previous studies demonstrate that this feature is peculiar of cross-linked HA hydrogels, and it is totally absent in not cross-linked HA dermal fillers that show microelements dispersed flake-like in aqueous solution [21].

### 2.2. Amplitude Sweet Test

The rheological properties of four HA-based dermal fillers were evaluated at isothermal conditions (25 – 37 °C) and the results are reported in Table 2 and Table 3, respectively. The amplitude sweep test makes it possible to determine Linear Viscoelastic Region (LVER) and demonstrates the HA hydrogels’ viscoelastic behavior. For each of them, elastic modulus (G’), viscous modulus (G’’), complex modulus (G*), tangent of the phase angle (tan δ) and complex viscosity (η*) were evaluated. A hydrogel shows a gradually decreasing value of the η* parameter at increasing strain values outside of the LVER. The viscosity represents the capacity of the sample to flow from the needle, while G’ is its ability in the LVER to resist deformation (strain), such as caused by facial movements and skin tension. The HA fillers’ capacity to resist deformation and to spring back (ability to return to the original shape) was demonstrated for these four cross-linked samples. The rheological parameters of a not cross-linked HA dermal filler were investigated in a previous study [21].

### 2.3. Frequency Sweep Test in Ramp Temperature Modality

**Figure 3:**
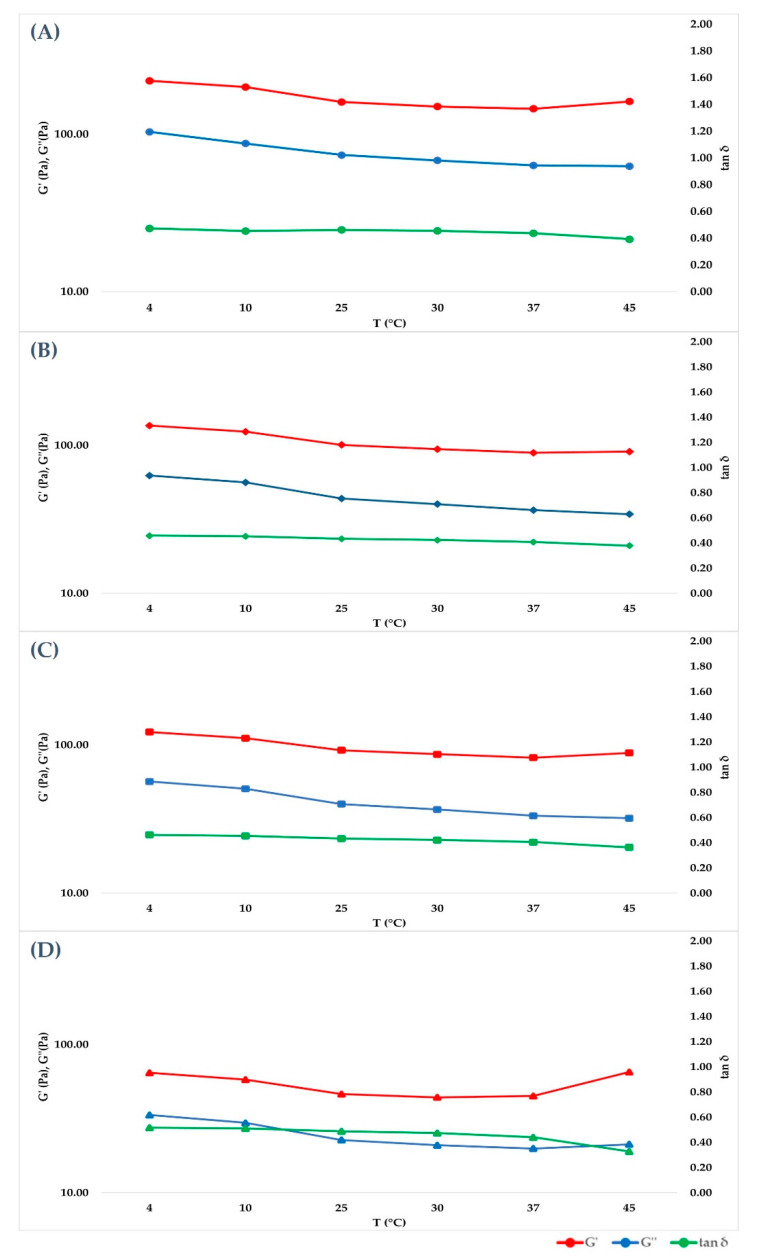
Cross-linked HA hydrogels show G’ higher than G” at every temperature point, showing the prevalence of elastic behavior on the viscous component. (**A**–**D**) are graphical representations of the cross-linked HA 28, 26, 24 and 22 mg/mL trends, increasing frequency.

**Figure 4:**
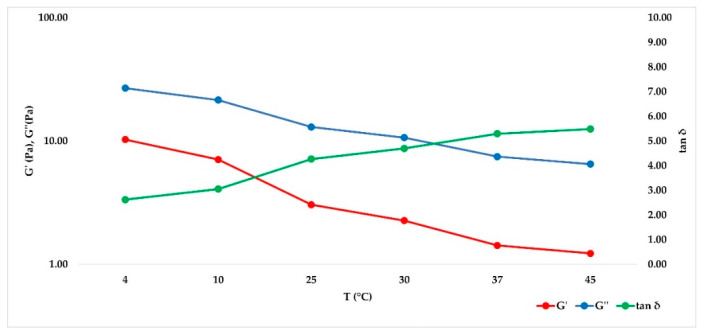
Not cross-linked HA trend of G’, G’’ and tan δ as a function of increasing temperature (4, 10, 25, 30, 37 and 45 °C).

**Figure 5:**
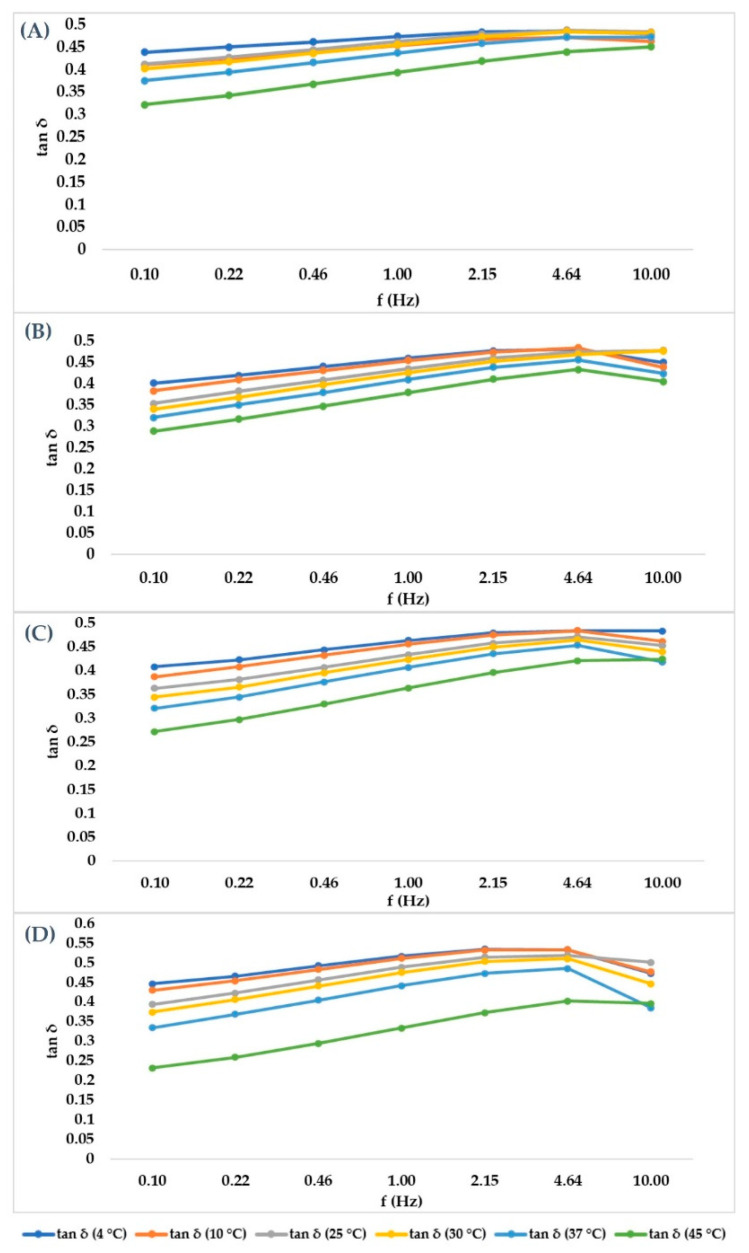
Cross-linked HA hydrogels graphical representation of the tangent of phase angle in order to summarize the trend of G’ and G’’, with tan δ given by the ratio of G’’/G’. (**A**–**D**) are graphical representations of the cross-linked HA 28, 26, 24 and 22 mg/mL trends, increasing frequency.

**Figure 6:**
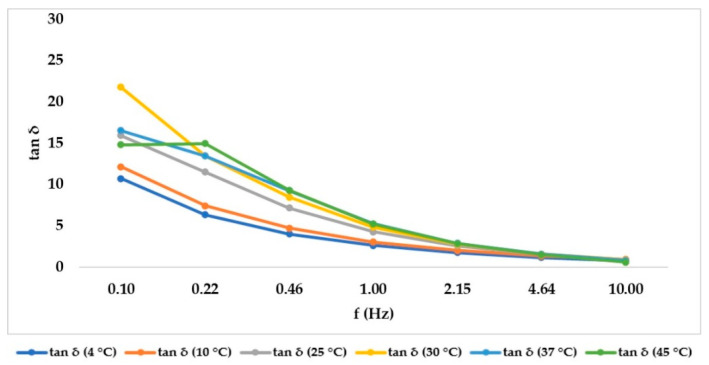
Not cross-linked HA hydrogels tan δ trend, increasing frequency, in order to summarize the trend of G ‘and G’’, with tan δ given by the ratio of G’’/G’.

After having identified the LVER in the amplitude sweep experiment, a 1% shear strain value was selected to carry out a Frequency sweep at different temperatures (4, 10, 25, 30, 37, 45 °C) in time course, without unloading the samples to reduce the errors. The purpose of the experiment was to mimic the behavior of the hydrogel when it is stored at different temperatures or after it has been injected in the living tissue. For cross-linked hydrogels, Figure 3 showed that G’ prevailed over G’’ in all cases, but both of their absolute values decreased when temperature was increased to 25 °C, then they remained constant. Tan δ, however, remained constant at every temperature set. As expected, the not cross-linked hydrogel presented a G’’ value that prevailed over G’ (behavior shown in Figure 4), underlining its liquid behavior. The values decreased within the entire temperature range but tan δ increased with temperature. Since tan δ is obtained by the ratio of G’’/G’, its trend summarizes the elastic modulus and the viscous modulus course. Data obtained at all temperatures and at all frequencies for each medical device investigated are showed in Figure 5 and Figure 6 for cross-linked HA-based dermal filler and not cross-linked HA hydrogel, respectively.

**Figure 7:**
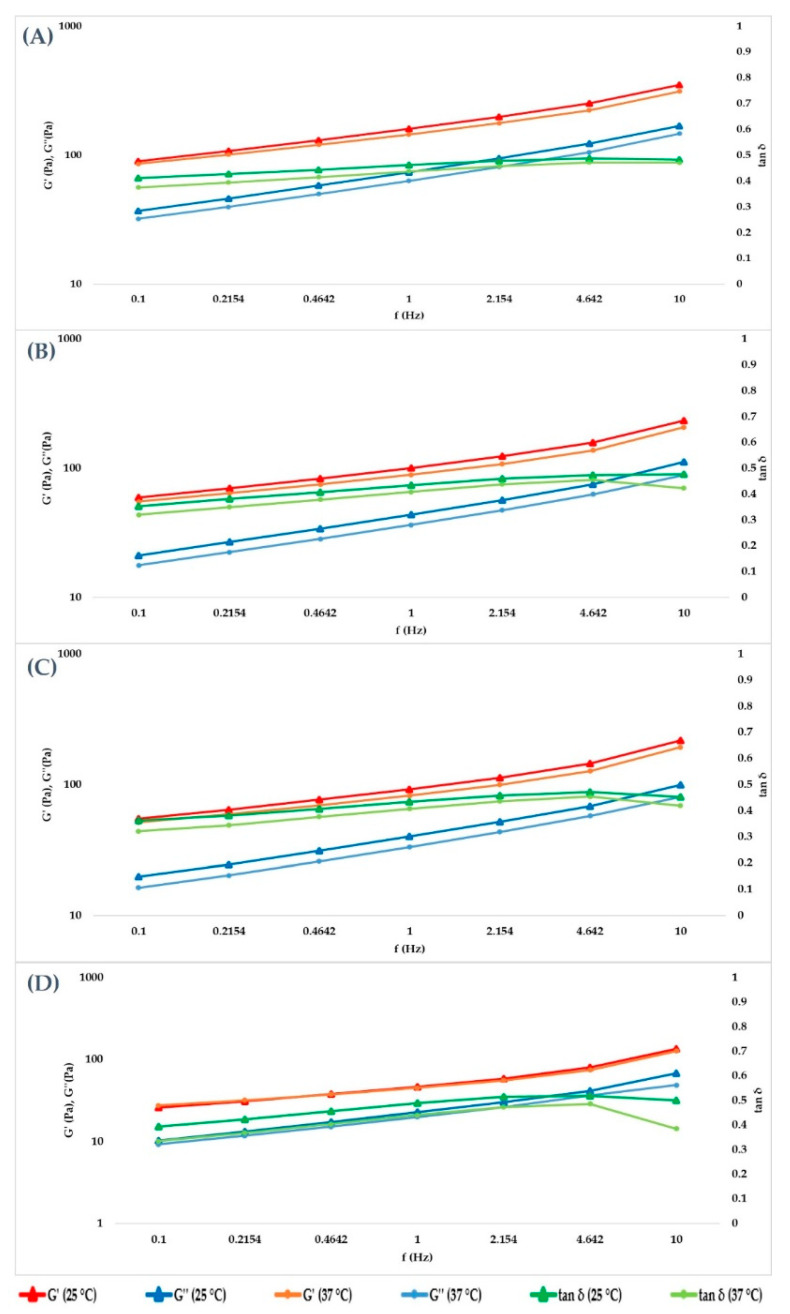
Cross-linked HA hydrogels G’, G” and tan δ. (**A**–**D**) are graphical representations of the cross-linked HA 28, 26, 24 and 22 mg/mL trends, at increasing frequency.

**Figure 8:**
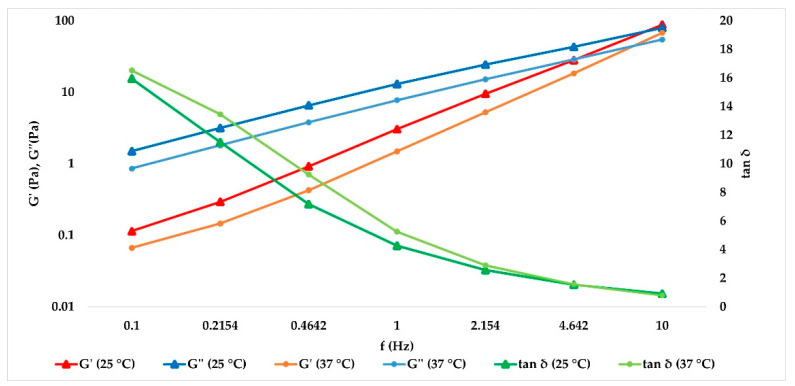
Not cross-linked HA demonstrates a clear prevalence of viscous behavior as the oscillatory frequency decreases both at 25 and 37 °C.

Data obtained at 25 and 37 °C were extrapolated, and the results are collected in Table 4 and Table 5, respectively. In Figure 7, the trend of the cross-linked fillers’ curves indicates that G’ and G’’ increase together with the frequency value. The storage modulus was always greater than loss modulus within the studied frequency range, both at 25 and 37 °C, for cross-linked hydrogels, and a tan δ value lower than unity was obtained, confirming the solid-like behavior of the hydrogels. For the not cross-linked filler, G’’ prevailed over G’, and they presented a crossover point close to 10 Hz, with tan δ values above unity (Figure 8). Therefore, the elastic nature of cross-linked gels prevailed over the viscous nature, while for the not cross-linked HA, the behavior was the opposite.

## 3. Materials and Methods

### 3.1. Hyaluronic Acid Fillers and Study Design

Five different HA-based dermal fillers, provided by Matex Lab S.p.A. (Brindisi, Italy), were characterized for their intrinsic matrix organization and rheological properties. Four of them were produced through a cross-linking technology combining HA and 1,4-butanediol diglycidyl ether (BDDE) as cross-linker agent. The quali-quantitative composition is reported in Table 1, highlighting the differences in HA concentration and presence/absence of cross-linking agent for each dermal filler.

### 3.2. Optical Microscopic Examination

For the microscopic investigation of the HA-based dermal fillers cross-linked with BDDE, 0.1 g of each hydrogel was placed into a 9-cm Petri dish containing 10 mL of milli-Q water and 30 µL of Toluidine Blue (Sigma-Aldrich, Missouri, USA) solution (1% *w/v* in water). The Petri dish was stirred slightly on a MS Orbital Shaker (Major Science, Saratoga, CA, USA) for 5 min, until the gel particles dissolution and staining [18]. Visualization was performed with an optical inverted microscope (VisiScope, VWR, Radnor, PA, USA) equipped with a digital camera, 5 plus, 5MP (Moticam Camera, Motic, Milan, Italy) and 10× magnification.

### 3.3. Amplitude Sweep Test for Linear Viscoelastic Region (LVER) Determination

Kinexus Plus Rheometer (Malvern Panalytical, Worcestershire, UK) was used for rheological analysis of HA hydrogel cross-linked with BDDE; data of the not cross-linked hydrogel 18 mg/mL were used as reference for the results interpretation [17]. This sequence is usually used to determine the linear viscoelastic region (LVER), where it is possible to subject the sample to a range of deformation without breaking its microscopic structure. G’ (elastic modulus), G’’ (viscous modulus) and tan δ (tangent phase angle) should be constant in LVER at increasing shear strain. Test was performed using a 20 mm plate–plate geometry (PU20 SR2467 SS), with a working gap of 1.00 mm, in isothermal conditions (25–37 °C), shear strain between 0.1 and 100%, and a frequency of 1 Hz. For each run, a fresh sample was loaded, and measurements were performed in triplicate. rSpace for Kinexus software (Malvern Panalytical, Worcestershire, UK) was used for the data processing.

### 3.4. Frequency Sweep Test with Increasing Temperature

Data obtained from amplitude sweep test were exploited in order to define a constant strain value to perform frequency sweep test in time-course, without unloading samples, and modulating progressively the temperature (4, 10, 25, 30, 37, 45 °C). An internal protocol was set up and the test was performed using a 20 mm plate–plate geometry (PU20 SR2467 SS), the working gap was set at 1.0 mm, and the analysis were carried out over a frequency range from 0.1 to 10 Hz, at 1% shear strain, in order to limit the gel’s system damage and to remain in the LVER (determined with the preliminary amplitude sweep test). Frequency sweep test is useful to determine G’, G’’ and tan δ. For each run, 5 min was required to cool or heat the cartridge, 5 min to cool or heat uniformly the hydrogel. Fresh sample was loaded, and measurements were performed in triplicate. rSpace for Kinexus software (Malvern Panalytical) was used for the data processing. Data obtained at 25 and 37 °C were extrapolated to predict the behavior of the filler in tissues, the stability of the product at rest or subjected to small external stress frequency. Tissue movements take place over several seconds; for this reason, Lorenc et al. concluded that 1 Hz is in the range of parameters relevant for physiological skin and facial movements [17,22].

## Figures and Tables

**Table 1:** Description of the HA-based dermal fillers provided by Matex Lab S.p.A., collected to investigate their microscopic structure and rheological properties.

Product	HA Content (mg/mL)	Cross-Linker
HA hydrogel 22 mg/mL	22	BDDE
HA hydrogel 24 mg/mL	24	BDDE
HA hydrogel 26 mg/mL	26	BDDE
HA hydrogel 28 mg/mL	28	BDDE
HA not cross-linked 18 mg/mL	18	Not cross-linked

**Table 2:** Rheological data of four HA hydrogel dermal fillers, provided by Matex Lab S.p.A., characterized with respect to their Linear Viscoelastic Region (LVER) at temperature of 25 °C and at a shear strain value of 1%. Data are presented as averages and relative standard deviation percentage (RSD %).

Product	G’ (Pa)	G’’ (Pa)	G* (Pa)	tan δ	η* (Pa s)
22 mg/mL	56.67 ± 1.98	26.03 ± 1.97	62.37 ± 2.62	0.46 ± 0.02	9.93 ± 0.42
24 mg/mL	97.27 ± 8.22	42.16 ± 2.99	106.02 ± 8.74	0.43 ± 0.01	16.87 ± 1.39
26 mg/mL	93.22 ± 4.91	41.65 ± 2.11	102.16 ± 4.15	0.45 ± 0.04	16.25 ± 0.66
28 mg/mL	120.57 ± 2.59	53.19 ± 0.10	132.60 ± 1.81	0.44 ± 0.01	21.10 ± 0.29

**Table 3:** Rheological data of four HA hydrogel dermal fillers, provided by Matex Lab S.p.A., characterized with respect to their Linear Viscoelastic Region (LVER) at temperature of 37 °C and at a shear strain value of 1%. Data are presented as averages and relative standard deviation percentage (RSD %).

Product	G’ (Pa)	G’’ (Pa)	G* (Pa)	tan δ	η* (Pa s)
22 mg/mL	56.99 ± 6.08	21.89 ± 1.77	61.05 ± 6.24	0.39 ± 0.02	9.72 ± 0.99
24 mg/mL	92.85 ± 1.51	37.01 ± 3.39	100.00 ± 0.92	0.40 ± 0.03	15.91 ± 0.15
26 mg/mL	95.26 ± 5.13	39.35 ± 0.94	103.04 ± 4.91	0.41 ± 0.02	16.40 ± 0.79
28 mg/mL	121.73 ± 5.59	52.86 ± 1.83	132.70 ± 5.88	0.43 ± 0.36	21.13 ± 0.93

**Table 4:** Rheological data obtained by the characterization of the five tested HA hydrogels at 25 °C and at a frequency value of 1 Hz. Data are represented as averages and relative standard deviation percentage (RSD %).

Product	G’ (Pa)	G’’ (Pa)	tan δ
22 mg/mL	46.39 ± 3.53	22.72 ± 6.91	0.49 ± 3.39
24 mg/mL	92.17 ± 6.12	39.96 ± 5.62	0.43 ± 4.73
26 mg/mL	100.75 ± 4.87	43.77 ± 7.13	0.43 ± 3.07
28 mg/mL	160.30 ± 8.62	73.98 ± 8.75	0.46 ± 4.92
Not cross-linked	3.04 ± 6.71	12.99 ± 10.22	4.27 ± 5.16

**Table 5:** Rheological results obtained by the characterization of the five HA hydrogel dermal fillers at 37 °C and at a frequency value of 1 Hz. Data are represented as averages and relative standard deviations percentage (RSD %).

Product	G’ (Pa)	G’’ (Pa)	tan δ
22 mg/mL	45.03 ± 5.77	19.90 ± 7.22	0.44 ± 4.46
24 mg/mL	82.13 ± 8.58	33.31 ± 5.79	0.41 ± 5.99
26 mg/mL	89.13 ± 6.78	34.49 ± 8.41	0.41 ± 2.92
28 mg/mL	145.20 ± 7.32	63.42 ± 7.14	0.44 ± 5.60
Not cross-linked	1.57 ± 6.08	6.73 ± 1.98	5.02 ± 7.33

## Data Availability

Data are included in the text; raw data are available from the corresponding author.
